# Cell-Mediated Immunological Biomarkers and Their Diagnostic Application in Livestock and Wildlife Infected With *Mycobacterium bovis*

**DOI:** 10.3389/fimmu.2021.639605

**Published:** 2021-03-04

**Authors:** Katrin Smith, Léanie Kleynhans, Robin M. Warren, Wynand J. Goosen, Michele A. Miller

**Affiliations:** Division of Molecular Biology and Human Genetics, Department of Science and Innovation-National Research Foundation Centre of Excellence for Biomedical Tuberculosis Research, Faculty of Medicine and Health Sciences, South African Medical Research Council Centre for Tuberculosis Research, Stellenbosch University, Cape Town, South Africa

**Keywords:** cell-mediated, cytokines, immunological biomarkers, livestock, *Mycobacterium bovis*, wildlife

## Abstract

*Mycobacterium bovis* has the largest host range of the *Mycobacterium tuberculosis* complex and infects domestic animal species, wildlife, and humans. The presence of global wildlife maintenance hosts complicates bovine tuberculosis (bTB) control efforts and further threatens livestock and wildlife-related industries. Thus, it is imperative that early and accurate detection of *M. bovis* in all affected animal species is achieved. Further, an improved understanding of the complex species-specific host immune responses to *M. bovis* could enable the development of diagnostic tests that not only identify infected animals but distinguish between infection and active disease. The primary bTB screening standard worldwide remains the tuberculin skin test (TST) that presents several test performance and logistical limitations. Hence additional tests are used, most commonly an interferon-gamma (IFN-γ) release assay (IGRA) that, similar to the TST, measures a cell-mediated immune (CMI) response to *M. bovis*. There are various cytokines and chemokines, in addition to IFN-γ, involved in the CMI component of host adaptive immunity. Due to the dominance of CMI-based responses to mycobacterial infection, cytokine and chemokine biomarkers have become a focus for diagnostic tests in livestock and wildlife. Therefore, this review describes the current understanding of host immune responses to *M. bovis* as it pertains to the development of diagnostic tools using CMI-based biomarkers in both gene expression and protein release assays, and their limitations. Although the study of CMI biomarkers has advanced fundamental understanding of the complex host-*M. bovis* interplay and bTB progression, resulting in development of several promising diagnostic assays, most of this research remains limited to cattle. Considering differences in host susceptibility, transmission and immune responses, and the wide variety of *M. bovis-*affected animal species, knowledge gaps continue to pose some of the biggest challenges to the improvement of *M. bovis* and bTB diagnosis.

## Introduction

*Mycobacterium bovis* infection and the resulting disease, commonly referred to as bovine tuberculosis (bTB), affects a broad range of species including humans, domestic animals, and wildlife (1, 2). Although *M. bovis*, as the name suggests, mainly affects bovids including cattle (*Bos taurus*), bison (*Bison bison*), African and Asian buffaloes (*Syncerus caffer* and *Bubalus bubalis*), it has been isolated from numerous other mammals, compromising animal and human health worldwide with reports of this species on all continents except Antarctica (3–5). Infection of wildlife further impacts livestock health due to the development of maintenance and spillover hosts within wildlife populations, including badgers (*Meles meles*) in the United Kingdom (UK), African buffaloes in southern Africa, and farmed and wild cervids in the United States (US) (6–8). In the UK and Ireland, *M. bovis* in badgers complicates bTB control efforts and similarly in the US, New Zealand, and Spain where deer, possum, and wild boar, respectively, are also recognized *M. bovis* reservoirs (4, 9). In the Americas, there are 15 wild species reported as infected with *M. bovis* (10). In South Africa, bTB is endemic in two major national parks, namely Kruger National and Hluhluwe-iMfolozi Parks (7). Moreover, *M. bovis* has been identified in more than 21 wildlife species in private and public sectors (11, 12).

In addition to the control challenges posed by infected wildlife species, bTB negatively affects wildlife-related industries, resulting in consequences for conservation, tourism, and game sales. Infection and disease in livestock and wildlife may lead to decreased productivity, trade restrictions, impacts on food security and zoonotic transmission, resulting in significant economic losses (5, 12). Bovine tuberculosis costs the global cattle industry alone 3 billion US dollars annually (13). In addition, ~5 million deer are farmed worldwide and bTB is the primary health threat across multiple species in this growing economic venture (14, 15). Despite effective bTB control measures in the US, states such as Michigan have endemic bTB in white-tailed deer (*Odocoileus virginianus*) populations that negatively affects the hunting and wildlife industry, in addition to the regular spillback affecting cattle (16, 17).

For these reasons, it is crucial to improve the detection, diagnosis and understanding of bTB across affected species to enable development of more effective and comprehensive control strategies. Early and accurate diagnosis of subclinical infection can inform more efficient management of affected animals. Alternatively, if associations can be accurately established between *M. bovis* infection or bTB disease and detectable host responses, limited available resources can be focused toward removing animals or populations that pose transmission risks to preserve those with higher economic, conservation or genetic value. However, the immune responses that arise from interactions between animal hosts and *M. bovis* are typically species-specific and particularly in the case of wildlife species, less well-characterized. Subsequently, validated diagnostic tests based on specific host responses to *M. bovis* infection and bTB are also scarce in wildlife.

The diagnostic standard for *M. bovis* detection, still in use despite being developed over a century ago, is the TST. The TST measures a host cell-mediated immune (CMI) response to mycobacterial purified protein derivative (PPD) antigens, either *M. bovis-*derived (PPD_b_) alone or together with *M. avium* (PPD_a_) for the single intradermal comparative tuberculin test (SICTT) (18, 19). In cattle, a meta-analysis of TST use in the UK and Ireland reported 100% specificity (Sp), in agreement with a Great Britain study displaying almost 100% Sp for standard and severe cut-off interpretations (20, 21). However, the sensitivity (Se) can range between 50 and 80%, with variations in Sp and Se between species and a lack of host-specific cut-off validation for all affected hosts (18, 21). A major confounding factor is the exposure to environmental mycobacteria in addition to vaccine strain antigens (for cattle in limited areas) that cause cross-reactions due to homology between antigenic peptides, demonstrated at gene and protein levels (19, 22, 23). In New Zealand, the TST displays reduced Se in deer exposed to environmental mycobacteria and is not recommended in herds with a high likelihood of *M. bovis* infection (14). When applied to fallow deer (*Dama dama*) in Texas (USA), the test had overall low Se and Sp, indicating minimal diagnostic value for this cervid species (14). There are also multiple cases of *M. bovis* crossing international borders in imported, infected deer due to negative TST results (14). In addition to variable test performance, the TST is subject to several limitations. The test is labor-intensive and logistically challenging in high income countries; in developing countries, this is exacerbated by restricted access to reagents and animals, veterinary capacity, and handling facilities (11, 24). An additional complication is management of cattle considered inconclusive reactors (IRs); in England and Wales, there were 3,755 IRs in 2015 alone (18). Animals in these herds may be infected, as suggested by the report that 21% of these herds had positive reactors when re-tested. In Ireland, between 11.8 and 21.4% of IRs slaughtered before a re-test were reported to be *M. bovis-*infected (18). However, movement of such herds is not typically restricted unless there is a recent history of TB, exacerbating *M. bovis* transmission risks when using the TST alone (18).

In attempting to address drawbacks associated with the TST in bovids, at least one ancillary test is recommended (25). One of the key focus areas of bTB research that has emerged is the discovery of new CMI biomarkers and consequently the development of CMI-based tests for the early detection of infection by pathogenic mycobacteria (26). Biomarker is a broad term defined as any indicator of pathogenic biological processes, either pathogen- or host-based (27, 28). The most common adjunct to the TST is the CMI biomarker-based interferon-gamma (IFN-γ) release assay (IGRA) (19, 29). However, despite improvements in Se for detecting *M. bovis-*infected animals, ancillary tests can introduce extra costs and logistical challenges. For example, IGRAs require that whole blood samples be processed within 8–24 h of collection, depending on the stimulation method, which is impractical if herds are located far from available laboratories (24). Moreover, IGRAs rely on a single measurement that is unable to discriminate *M. bovis* infection from bTB disease and further, may fail to diagnose early infection when blood is stimulated with more specific mycobacterial peptides instead of PPDs (29, 30). To overcome this, a combination of biomarkers may be used to improve diagnostic Se. For example, progress has been made in enhancing the detection of bTB infection in cattle by combining IGRAs with the simultaneous measurement of antigen-specific interleukin-1β (IL-1β) or IFN γ-induced protein 10 (IP-10) (29–31). A systematic review by Domingos et al. (10) of *M. bovis* diagnostic methods in wildlife species also suggested the application of at least two tests to aid the detection of infection and potentially different disease stages.

A primary goal for accurate *M. bovis* detection is the early identification of a maximal number of infected animals for more effective control measures. The investigation of candidate biomarkers for *M. bovis* infection and bTB is ongoing, as discussed below. However, if current biomarker research could be further advanced to differentiate infection from bTB disease, this would enable a focus on detecting stages in which the animal is potentially infectious, improving the elimination of major transmission risks or preventing infected individuals from transitioning to a diseased state. The primary *ante mortem* diagnostic tools in livestock and wildlife rely on the development of antigen-specific CMI responses to *M. bovis* (30, 32, 33). Therefore, this review will focus on current understanding and knowledge gaps regarding the host response to *M. bovis* infection and the use of host CMI biomarkers for the improved understanding and diagnosis of *M. bovis* infection and bTB disease.

## Host Responses to *Mycobacterium bovis*

With the availability of bovid host and *M. bovis* genomes, the understanding of host responses to *M. bovis* has continued to develop (24). Immunity, in brief, comprises the innate and adaptive immune systems. The adaptive immune response has two distinct components, namely humoral and cell-mediated immunity, of which the latter is vital for host protection against *M. bovis* infection. All the major T lymphocyte subsets have demonstrated involvement in the anti-mycobacterial immune response in cattle (34). In *M. bovis-*infected cattle, CD4^+^ T cells produce IFN-γ for activation of macrophage anti-mycobacterial functions whereas CD8^+^ T cells are involved in the lysis of infected cells (34). The development of a T helper type 1 (Th1) -based CMI response, resulting in production of cytokines and chemokines such as tumor necrosis factor-α (TNF-α), IL-12, IL-6, and IFN-γ by dendritic cells and macrophages, is considered essential for the control of mycobacterial infection (35). Cytokine production is activated by pathogen antigen presentation to immune cells, and disease progresses when these pathways are disrupted (36). Cytokines play a critical role in determining the characteristic immune responses to infection; the balance between Th1 and T helper type 2 (Th2) functions is often studied from the perspective of their cytokine profiles, each of which fluctuate throughout disease progression (35). Cytokine function can vary from pro-inflammatory, which promotes activation of cells to kill mycobacteria, to anti-inflammatory, which reduces the pro-inflammatory response to control and prevent tissue damage by necrosis (37). In the primary stages of mycobacterial infections, Th1-dominant cytokine responses develop (36). The Th1 immune response is critical for defense against intracellular pathogens, most notably through the production of IFN-γ (34). However, a shift from Th1- to Th2-based responses is typically observed as disease develops, with a shift in CMI responses along with increasing humoral (B cell) responses (38). The Th2 response is associated with the production of cytokines including IL-4, IL-5, and IL-10 with studies suggesting that the Th1/Th2 balance is critical for determining bTB disease progression and outcomes (39). Therefore, any changes in immune responses during the course of infection are likely to affect the diagnostic performance of immunological assays.

Although early detection of *M. bovis* infection is primarily dependent upon the measurement of CMI responses in many species, humoral responses have also been used to diagnose bTB (35, 40–42). In certain species, for example elephants (*Loxodonta africana* and *Elephas maximus*) and suids, anti-mycobacterial antibodies can be detected during early infection (43–46). In species such as cattle and lions (*Panthera leo*), on the other hand, antibody detection usually occurs only after bTB disease progresses and the immune responses shift toward a Th2 profile (41, 47). However, the application of serological tests requires insight into the humoral immune profile of specific species to determine the differences in immunodominant antigen recognition and response kinetics (48, 49). Due to a paradigm that early immune responses are cell-mediated, this has been the primary focus of bTB research in animals to date although further studies on comparative immunobiological responses in different species are warranted.

In cattle and some other species, a CMI-dominated early and specific response to *M. bovis* infection is observed with proliferation of antigen-specific T lymphocytes, secretion of the regulatory cytokine IL-2, and release of pro-inflammatory cytokines including IFN-γ. These responses have been reported, for example, in two studies by Corner et al. (50) and Gormley and Corner (51) that investigated CMI responses to *M. bovis* in experimentally infected badgers, one of the few relatively well-studied non-bovid species. The earliest CMI immune responses were observed 3 weeks post infection (WPI) in the animals infected with the highest *M. bovis* dose; these animals also demonstrated the most consistent CMI responses of peripheral blood mononuclear cells (PBMCs) to *M. bovis* PPD (PPD_b_) stimulation. The overall CMI responses were also positively associated with pathological changes, i.e., the presence of gross lesions, detected *post mortem*. On the other hand, the antibody responses in badgers were only sporadically detected.

Functional genomics and proteomics have enabled a better understanding of CMI responses to *M. bovis* infection (52). Studies typically focus on the *in vitro* assessment of gene expression profiles in different cells or tissues, isolated after infection with *M. bovis* or related strains, and *ex vivo* analyses aimed at identifying cytokine gene expression signatures for the detection of *M. bovis-*infected animals. Wedlock et al. (53) compared gene expression in primary bovine alveolar macrophages (AMs) infected with a virulent *M. bovis* strain and an attenuated version. Although both strains grew at comparable rates, the results suggested a 45% difference in gene expression between the two infection groups. Of the 10 most differentially expressed genes, with the virulent strain inducing higher levels of expression, seven of these were chemokines with *IL-8* and monocyte chemoattractant protein (MCP)-1 (*CCL2*) having the greatest expression. The conclusion was that AMs infected with virulent *M. bovis* displayed a more dominant pro-inflammatory gene expression profile than those infected with the attenuated strain. Another study using a similar method observed lower expression of chemokines from AMs of *M. bovis-*infected cattle than *M. tuberculosis*-infected cells, highlighting a possible mechanism employed by *M. bovis* to circumvent activation of the host's chemotactic response, thereby evading killing (54).

Although the host immune responses during *M. bovis* infection and disease remain less understood than for *M. tuberculosis*, recent studies have begun to shed more light on this complex pathogen-host interplay. However, susceptibility to infection, routes of infection and disease progression can vary significantly between the wide range of host species that are affected. Hence, it would be expected that immune responses and therefore, specific diagnostic biomarkers may also differ, impacting advances in host biomarker discovery and application (36).

## Host Diagnostic Biomarkers of Pathogenic Mycobacterial Infection

A suitable diagnostic biomarker or multiple marker biosignature, for infection or disease, is a host- (or pathogen-) specific molecule/protein that is associated with the underlying pathological process (28, 55). Identification of biomarker-based assays that can enable more accessible, affordable, and efficient diagnosis has been a high priority for TB and bTB diagnostic research in recent years (28, 56). Considering the World Health Organization's target product profiles, that define the performance and operational characteristics of suitable tests, it is expected that non-DNA markers are more likely to meet practical and cost targets (28, 57). Non-DNA methods can be performed without advanced instrumentation, utilize more easily accessible samples such as blood and serum, and are generally more affordable than DNA-based tests (27, 28). Hence, the focus of current bTB diagnostic research is host biomarkers with an emphasis on cytokine/chemokine (chemotactic cytokine) proteins and antibodies, in addition to the detection of cytokine RNA expression (5, 24, 58).

Critical barriers to progress regarding biomarker discovery include the lack of standardization in specimen collection methods and a reliance on convenient or opportunistic samples, in addition to inadequate handling and storage of samples which can drastically alter detectable biomarker levels (51, 59). Another common limitation is suboptimal statistical power due to low sample numbers, hence, associations between biomarkers and potential disease states are challenging to investigate and prove (59). A biomarker should address a definitive question related to a particular state, or the prognosis, of pathogenic mycobacterial infection in individuals or populations (55). However, the stages of TB that have been elucidated from human research are complex and include infection, incipient or subclinical or active disease, and latency; moreover, these states are not regarded as fixed but instead constitute a dynamic spectrum that may be affected by multiple factors, many of which remain poorly understood (55, 60).

For *M. bovis* infections in animals, insight regarding the mycobacterial disease spectrum is even more limited (36, 51, 60). In general, *M. bovis* infection in animals is often not detectable until disease is advanced. In addition, clinical signs are often non-specific such as weight loss, decreased milk production (in dairy cattle), and occasionally coughing or other respiratory signs (11, 61). Chronic disease can progress to mortality over the course of months to years. The hallmark lesion of bTB in cattle is the granuloma that can be found in the lung, lymph nodes and potentially other organs (62). Granuloma stages have been well-described in cattle and are based on morphological characteristics, i.e., degree of necrosis and mineralization, and presence of a connective tissue capsule (63). However, *M. bovis* disease development and pathological lesions can vary widely between species, with cavitary pulmonary lesions instead of granulomas in goats and gastrointestinal infection observed in predators, for example lions (64–66).

To date, the only method of determining an animal's true infection status is comprehensive *post mortem* examination and a battery of mycobacteriological, immunological, histopathologic, and molecular tests (19). Accurate *ante mortem*, in-field reference tests for infection and/or active bTB disease are lacking (56). Current understanding of the pathogenesis of *M. bovis* infection is also confounded by the multitude of susceptible animal hosts and the complexity of the host-pathogen interactions (36, 51). Although naturally-infected animals are useful in the investigation of differences between infection and disease, experimental models illuminate disease progression kinetics and immune responses initiated from a fixed point in time with a set dose, generating the insight necessary for diagnostic test development (67, 68). A major limitation for *M. bovis* and bTB diagnostic research is the lack of clarity regarding case definitions for infection vs. active disease and, whether latent infections exist (51, 60, 69, 70). Considering the various limitations outlined above, the fitness for purpose criteria [as defined by the World Organization of Animal Health (OIE)] are critical for the validation and application of diagnostic bTB biomarkers in animals and should be applied on an individual species basis.

The *ante mortem* diagnosis of bTB in animals primarily relies on the detection of host CMI responses, most commonly with the use of protein and gene expression assays (5). Although the most widely used ancillary test, IGRA, in addition to the TST, can produce false negative results as the CMI responses required may diminish in advanced disease stages (anergy), while the humoral response increases (35, 69). Furthermore, IGRAs demonstrate limited Se in early infection stages and in general, bTB diagnostic assays are unable to distinguish infection from disease (13, 21, 25, 58, 71). On the other hand, a recent study by Bernitz et al. (72) observed that levels of IFN-γ and IP-10 in incubated unstimulated whole blood were elevated in infected buffaloes with observable pathological changes consistent with bTB in comparison to uninfected controls. Furthermore, increased IFN-γ significantly correlated with increasing severity of pathological changes in the infected buffaloes, consistent with observations of associations between antigen-stimulated IFN-γ and bTB pathology in cattle and badgers, demonstrating the potential for these cytokines to be used as indicators of bTB disease (72–74).

A limitation of cytokine/chemokine protein assays can be the inability to detect high levels of the biomarker target due to, for example, binding saturation limits; therefore, mRNA responses and their detection may provide a more robust alternative (5). There are several studies that investigate cytokine/chemokine RNA expression to achieve diagnostic test objectives. However, another purpose of biomarkers is to improve the fundamental understanding of disease pathogenesis, which is often the focus of bTB biomarker RNA expression research, for example studies that characterize the immunological profile of *M. bovis-*infected cattle (experimental or natural) by measuring cytokine/chemokine mRNA expression at various time points during infection and/or disease (35, 39, 53, 55, 75–77). Immune response changes during bTB disease progression, particularly in terms of anti- and pro-inflammatory cytokine/chemokine profiles, can signify disease outcomes and hence play a role in the diagnosis and control of infection (35). The CMI response is the dominant immunological response to *M. bovis* (in most studied species), responsible for the killing and elimination mechanisms in addition to formation of characteristic granulomas against this intracellular pathogen (69). The following section will further describe some of the CMI biomarkers of infection and bTB disease in animals.

## Cytokine/Chemokine Biomarkers of *Mycobacterium bovis* Infection

**Figure 1:**
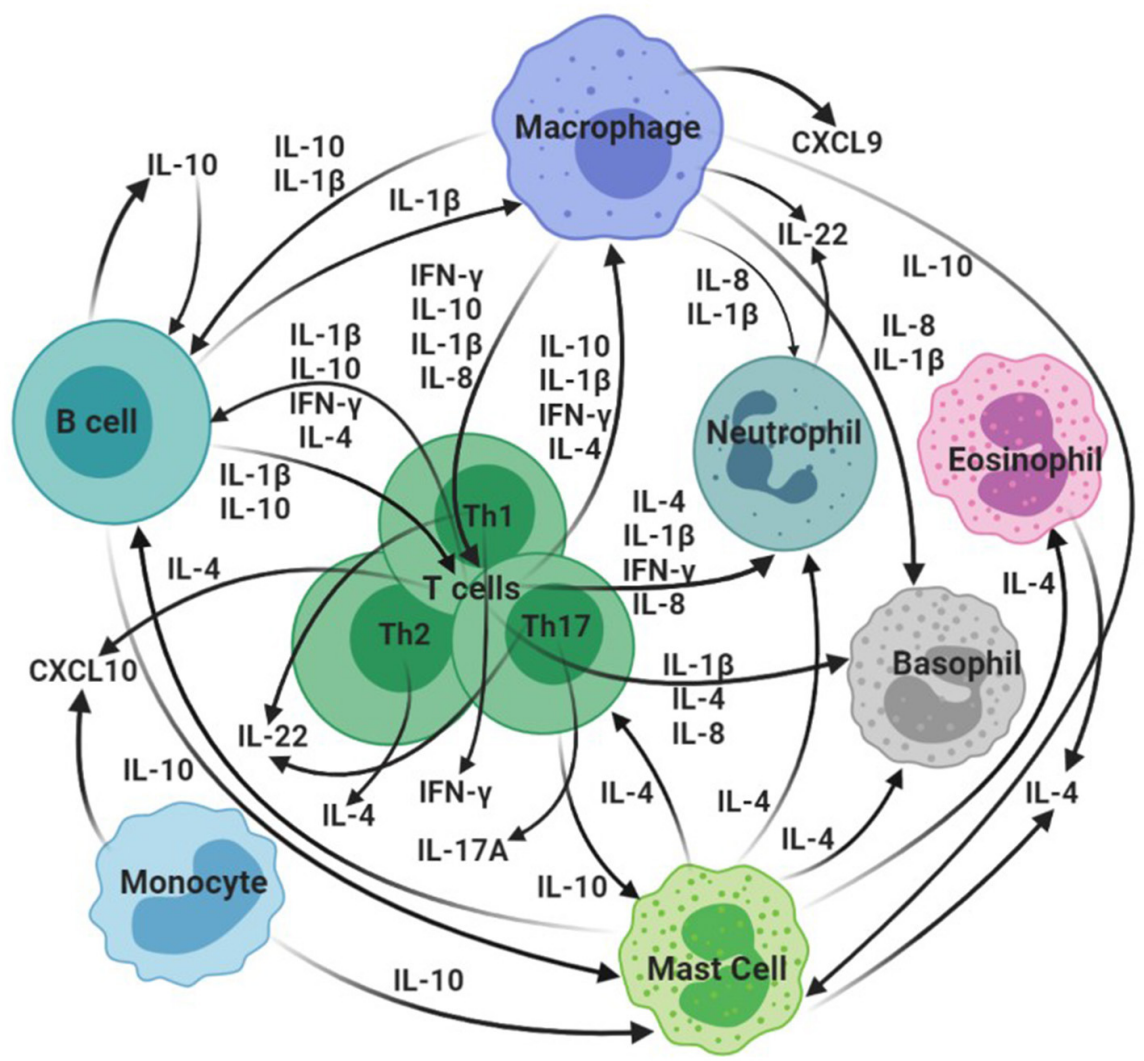
Overview of hypothesized interactions of selected cytokines/chemokines identified as bTB biomarkers in domestic and wild animal species. CXCL10: IP-10. Created in BioRender.com.

Several promising cytokine/chemokine biomarkers of *M. bovis* infection and disease in domestic and wildlife species have been revealed in recent years, in addition to the widely recognized host biomarker IFN-γ. These include, although are not limited to, IP-10, IL-1β, IL-4, IL-8, IL-17A, *CXCL9*, IL-10, and IL-22 (37, 40, 78–84). Both protein and gene expression assays have been used to assess biomarker levels in different species and have facilitated identification of additional targets for diagnostic development (5). This section will describe select current and candidate CMI biomarkers of *M. bovis* infection in domestic and wild animal species, as summarized in Figure 1 and Table 1.

**Table 1:** Cell-mediated immune cytokines that have demonstrated potential as bovine TB biomarkers that distinguish *M. bovis-*infected from uninfected domestic and/or wild animal species.

**Cytokine**	**Species**	**Reference(s)**
IL-4^*^	Cattle, white-tailed deer	(37, 75, 76, 78)
IL-8^*^	Cattle	(39, 79, 84)
IL-17A^*^	Cattle	(80, 81, 85–89)
IL-22^*^	Cattle	(5, 25, 80, 88, 90–93)
IL-10^*^	Cattle, goat	(35, 37, 94–97)
IFN-γ^*^	Multiple (see Table 2)	(72, 74)
IP-10^*^	Warthog, African buffalo, cattle	(29, 30, 72, 98, 99)
IL-1β	Cattle	(5, 31, 82, 91)
CXCL9	Cattle, lion, spotted hyena, cheetah, warthog	(5, 25, 77, 92, 100–103)

IL, interleukin; IFN-γ, interferon-gamma; IP, IFN-γ-induced protein;

* *have also shown potential to distinguish between different bTB states i.e., early infection, active disease*.

### IFN-γ

The cytokine IFN-γ is a vital mediator of macrophage activation, amplifying macrophage cytokine release in response to *M. bovis* and playing a critical role in host protection and pathogen control (74, 104). The utility of various cytokine release assays based on IFN-γ (IGRAs) has been demonstrated in cattle, leading to the acceptance of IGRAs as an adjunct to the TST in European legislation, and some countries outside of the European Union (61, 105). The first bTB *in vitro* IGRA was developed in the 1980's in Australia and is now used globally (106). Studies on *M. bovis* experimentally- and naturally-infected cattle have demonstrated the ability of IGRAs to detect a positive CMI response from as early as 2 weeks post-infection and often earlier than detection by the SICTT (34, 78, 107, 108). The most used IGRA platform in worldwide bTB control programmes is the Bovigam® which uses PPD_b_ and PPD_a_ stimulatory antigens (71). The Bovigam® IGRA is also effectively applied to *M. bovis* detection in goats and another PPD-based IGRA has been used in pigs (109, 110).

Currently, the primary ancillary method for *M. bovis* detection in African buffaloes is the IGRA, based on either PPD stimulation or the QuantiFERON® TB Gold (QFT) system that stimulates whole blood with *M. bovis/M. tuberculosis-*specific antigens early secretory antigenic target 6 (ESAT-6) and culture filtrate protein 10 (CFP-10) (32, 111). Thereafter, species-compatible IFN*-*γ enzyme-linked immunosorbent assays (ELISAs) are used to detect the biomarker. Validated IFN*-*γ ELISAs for buffaloes include the Bovigam® assay and Mabtech in-house, Cattletype®, and commercial Mabtech bovine IFN*-*γ ELISAs (33, 111, 112). There are several other wildlife species for which IGRAs to detect *M. bovis* infection have been developed, as summarized in Table 2.

**Table 2:** Summary of interferon-gamma (IFN-γ) release assays (IGRAs) employed in domestic and wild animal species for *M. bovis* detection and bTB diagnosis.

**Species**	**Reference(s)**	**Detection method**	**Stimulatory antigen(s)**
Buffalo (*Syncerus caffer*)	(32) !!break (33) !!break (111)	Bovigam® IGRA !!break QFT/Mabtech ELISA !!break QFT/Cattletype® ELISA	PPD !!break!!break EC !!break!!break EC
White rhino (*Ceratotherium simum*)	(113)	QFT/Mabtech ELISA	EC
Wild dog (*Lycaon pictus*)	(114)	QFT/R&D Quantikine ELISA	EC
Badger (*Meles meles*)	(115)	IGRA (in-house)	PPD/EC
Alpaca (*Vicugna pacos*)	(116)	Mabtech ELISA	PPD/EC
Red deer (*Cervus elaphus*)	(117)	IGRA (in-house)	PPD/EC/Rv
White-tailed deer (*Odocoileus virginianus*) !!break Reindeer (*Rangifer tarandus*) !!break Sambar deer (*Cervus unicolor*)	(118)	Cervigam™ ELISA	PPD
Cattle (*Bos taurus*)	(21) (Meta-analysis)	Various	PPD/EC
Goat (*Capra hircus*)	(110)	Bovigam® IGRA	PPD
Pig	(109)	IGRA (in-house)	PPD

*PPD, purified protein derivative (bovine/avian); EC, ESAT-6/CFP-10; Rv, Rv3615c/Rv3020; QFT, QuantiFERON®-TB Gold stimulation platform*.

Using ESAT-6/CFP-10 (EC)-stimulated whole blood (QFT platform), IFN*-*γ ELISAs have been modified for use in white rhinoceros (*Ceratotherium simum*) and wild dog (*Lycaon pictus*) (113, 114). Using both PPD and EC stimulatory antigens, IGRAs have also been used in badgers, with good Se and Sp, alpacas (*Vicugna pacos*), and red deer (*Cervus elaphus*) for which Rv3615c and Rv3020 antigens were also used (115–117). The Cervigam™ IFN-γ ELISA (developed for red deer), used with plasma from PPD-stimulated whole blood, has also displayed promise for bTB diagnosis in white-tailed deer in addition to reindeer (*Rangifer tarandus*) and sambar deer (*Cervus unicolor*) (118).

Although IFN-γ has proved invaluable for several species as a biomarker of *M. bovis* infection, in other species, including lions and warthogs (*Phacochoerus africanus*), it does not appear to be as useful (83, 98). It has also been observed that depending on the infection phase, IGRAs (and the SICTT) may fail to detect *M. bovis-*infected animals, which could be due to either very early infection (when a response detectable by the IGRA is still forming) or a depressed (anergic) immune state occurring in the later stages of bTB (119, 120). In addition, there may be individual or species differences in immune responses, resulting in failure of IGRAs to detect infection. For example, suids and New World camelids have robust humoral responses to mycobacterial infection and although detection of IFN*-*γ has been reported, these assays are not commonly used as diagnostic tests in these species (46, 100, 109, 116, 121). Moreover, IGRA results can be confounded by co-infections with environmental strains, vaccination (in some domestic species) and, although contended, possible interference by previous testing including the SICTT (13, 69). The host immune response to mycobacteria is naturally linked to disease progression yet bTB generally presents with extended and advanced, poorly described disease stages, posing an additional challenge for the extension of IFN*-*γ-based diagnostics to distinguish infection from active disease (120, 122). However, correlations between IFN*-*γ and *M. bovis* pathology have been preliminarily detected in badgers and buffaloes (72, 74). Moreover, investigating the associations between cytokine responses at both a gene and protein level, alongside host-specific pathological changes, can provide more insight on the diagnostic potential of IFN*-*γ and additional biomarkers (5, 63, 75, 94, 123, 124).

### IP-10

The IFN*-*γ*-*induced chemokine IP-10, expressed by lymphocytes and monocytes, is produced at high levels in humans, cattle and African buffaloes, up to 100-fold more than IFN*-*γ, following infection with tuberculous mycobacteria (99, 125, 126). It has a role in delayed type hypersensitivity reactions and antigen-induced levels of IP-10 have shown promise for early detection of *M. bovis* infection in animals that may be negative on other tests (such as IGRAs) (30, 98, 125, 126).

Considering the potential for IP-10 to be a more sensitive marker than IFN*-*γ, there are still relatively few studies in bTB-affected species. Using a Kingfisher Biotech (St Paul, MN, United States) bovine IP-10 ELISA and QFT whole blood stimulation, a significantly higher antigen-specific IP-10 response was able to distinguish *M. bovis-*infected from culture negative warthogs (98). The same has been shown in African buffaloes using the same platform, and IP-10 has demonstrated high test Se in this species (30, 40). Furthermore, higher levels of IP-10 in incubated samples without antigen stimulation have been correlated to the detection of *M. bovis* pathology in infected buffalo populations (72). Using the same assay as for buffaloes, Parsons et al. (99) showed a strong correlation between IP-10 and IFN-γ release, and the robustness of IP-10 as a biomarker of *M. bovis* infection in cattle. Waters et al. (127) also observed antigen-specific IP-10 mRNA responses in PBMCs from cattle, starting at 29 days after *M. bovis* challenge, that were highly correlated to IFN-γ mRNA levels; and Palmer et al. (5) confirmed the potential of IP-10, with mRNA isolation and protein release from whole blood, for bTB diagnosis in cattle. However, similar to what Parsons et al. (99) reported, high levels of IP-10 in unstimulated plasma from both infected and uninfected cattle were observed for some individuals. The parallel measurement of IP-10 with IFN-γ has also demonstrated the potential to augment detection of *M. bovis-*infected cattle and buffaloes, highlighting the benefit of host biomarker signatures for enhanced bTB diagnosis (29, 30).

### IL-1β

IL-1β is one of the multiple cytokines secreted, primarily by innate immune cells such as monocytes and macrophages, with IFN-γ, to orchestrate an immune response toward mycobacterial infection and is viewed as one of the major pro-inflammatory cytokines (82, 128). Limited biomarker studies beyond cattle include the detection of IL-1β mRNA and protein in heat-inactivated *M. bovis-*immunized red deer compared to controls and, in a separate study of red deer, an observed association with disease progression after challenge with an *M. bovis* field strain (95, 129). Jones et al. (31) found IL-1β cytokine levels to be much higher in *M. bovis-*infected compared to uninfected cattle, using whole blood stimulated with either PPDs or EC. Elnaggar et al. (82) found similar results when stimulating blood with EC antigens, with significant differences observed between the infected group, and both non-tuberculous mycobacteria-exposed and uninfected control cattle. Palmer et al. (5) investigated IL-1β expression in whole blood stimulated with EC and Rv3615c, showing significantly higher levels in the *M. bovis-*challenged cattle, compared to uninfected controls, at 5-, 8-, and 12-weeks post infection (WPI). Rusk et al. (91) demonstrated significant upregulation of IL-1β by *M. bovis-*specific T cells, isolated from stimulated PBMCs, by transcriptomics analysis and T cell/macrophage co-cultures using experimentally infected calves. Finally, the study by Jones et al. (31) applied IFN-γ and IL-1β assays in parallel to observe a 5% increase in Se without any loss of Sp, using the EC antigens, when compared to measuring IFN-γ alone; this reiterates the utility of biomarker signatures for more efficient diagnosis. Although not used in the context of mycobacterial infection, IL-1β and IL-8 sequences have been reported for the Asian elephant (*Elephas maximus*) and may provide a foundation for investigating antigen-specific immune responses using gene expression assays (130).

### IL-4

A characteristic indicator of the Th2 immune response to mycobacteria, IL-4 plays an anti-inflammatory role such as controlling tissue damage by down-regulating pro-inflammatory responses (37). Rhodes et al. (78) investigated IL-4 cytokine expression in PBMC culture supernatants, after stimulation with PPDs or EC, using experimentally and naturally *M. bovis*-infected cattle. The overall IL-4 response, in comparison to IFN-γ, was delayed with activity peaking at 6–8 WPI. Challenge with a low dose of *M. bovis* caused a reduced IFN-γ response although a specific IL-4 response remained evident. In naturally infected cattle, increased IL-4 differentiated these animals from uninfected controls with Se equivalent to that of IFN-γ (78). These findings are similar to a study by Blanco et al. (39) in which IL-4 expression in PBMCs was elevated in five of nine naturally infected cattle, compared to controls.

Two studies by Thacker et al. (75, 76) on experimentally infected white-tailed deer and cattle, respectively, also found that increased IL-4 gene expression distinguished *M. bovis-*infected from uninfected animals when investigating PBMCs stimulated with PPDs or EC. The highest IL-4 levels in deer were at 12, 16, or 24 WPI; for cattle, the peak was earlier (at 4 WPI) with a decline thereafter, similar to the Rhodes et al. (78) study. In the deer, *M. bovis* infection resulted in consistently more IL-4 production in animals with less pathological changes compared to the high pathology group. In cattle, however, IL-4 expression was higher in the high pathology group overall, regardless of stimulus and the low pathology group was indistinguishable from the uninfected group. In contrast, Widdison et al. (37) assessed infected cattle 16 WPI, when the acute infection phase was more controlled and chronic disease was starting to develop. In this study, a significant reduction in IL-4 with an increase in the IFN-γ/IL-4 ratio was observed in *M. bovis-*challenged animals. Moreover, there was a significant negative correlation between IL-4 expression, and both lymph node scores and the number of mycobacteria isolated. This supports the hypothesis of a conversion from Th1- to Th2-dominated responses in the first 3 months post *M. bovis-*infection in cattle (76). These findings also suggest that species-specific patterns of cytokine responses need to be defined.

In summary, it appears that the observed delay of the IL-4 response relative to IFN-γ corresponds to the anti-inflammatory role of IL-4 in *M. bovis* infection (37, 78). Two of the cattle infection studies demonstrated early peaks in IL-4 expression followed by rapid decreases, while IFN-γ responses remained detectable when lesion development would be expected to begin (75, 78). This suggests that IL-4 may reduce IFN-γ-induced pathology and does not compromise the protective response, although results have suggested that the switch from Th1 to Th2 responses may occur later than 3 months PI (76). Although not yet investigated outside bovids and cervids, the potential for IL-4 to distinguish infection states warrants further investigation.

### IL-8

The precise role of the chemokine IL-8 in TB has not been fully elucidated, although studies have demonstrated IL-8 binding to tubercle bacilli and interactions with the pathogen that appear to enhance mycobactericidal characteristics of macrophages and neutrophils (131). Increased IL-8 is also required for granuloma formation (84).

Widdison et al. (79) investigated RNA expression of IL-8 in lymph node tissue from *M. bovis-*challenged cattle. The infected cohort displayed lesions at necropsy, representing a well-established infection stage and the same cohort displayed significantly elevated IL-8 expression. Widdison et al. (79) also observed a positive correlation between IL-8 expression levels and lesion severity, together with the bacterial load, in the lymph nodes examined. In contrast, Blanco et al. (39) observed decreased IL-8 expression in naturally infected vs. healthy cattle. The lack of IL-8 upregulation, together with the observed Th1 cytokine profile in PPD_b_ stimulated PBMCs, was indicative of active infection (39). In humans, low IL-8 mRNA expression, in combination with other markers, allows differentiation between active and latent TB (132).

A more recent study by Gao et al. (84) investigated naturally *M. bovis-*infected cattle, further characterized by a nested PCR, on *M. bovis* bacteria shed into nasal exudates, that could identify animals that posed higher transmission risks (i.e., PCR-positive and at a more advanced bTB stage). An IL-8 assay was performed with PPD_b_- and EC-stimulated whole blood from cohorts of infected/PCR-negative (bTB_PCR−N_), infected/PCR-positive (bTB_PCR−P_), and uninfected cattle. Both stimuli resulted in significantly increased IL-8 in both *M. bovis-*infected cohorts compared to the uninfected cattle. Interestingly, unstimulated IL-8 was significantly higher in the bTB_PCR−N_ cohort than both the bTB_PCR−P_ and uninfected cohort. The concentrations of PPD-stimulated IL-8 were also positively correlated with IFN-γ and were higher than the levels of IFN-γ, IL-17A, and IP-10. In addition, PPD-stimulated IL-8 production was able to better discriminate *M. bovis-*infected animals from uninfected animals than IP-10 and IL-17A and showed good agreement with the TST and IGRA with a relative Se and Sp of >90 and >98%, respectively (84). Although relatively less reported, results obtained from studies of IL-8 in cattle suggest an important role of this chemokine in *M. bovis* infections (84). However, further research to determine the potential of IL-8 as a bTB biomarker is still required.

### IL-17A

The pro-inflammatory cytokine IL-17A (IL-17), produced by Th17 lymphocytes, has been identified as a primary effector cytokine necessary for detection and clearing of tubercle bacilli (133). Studies of IL-17A in bTB have demonstrated its role in immunity against mycobacterial infection, in addition to participation in granuloma formation (85). Blanco et al. (86) studied *IL-17A* mRNA expression in PPD_b_-stimulated PBMCs from experimentally infected cattle and found that animals with macroscopic bTB lesions developed higher *IL-17A* expression compared to cattle without lesions, with statistical significance at 60- and 90-days PI (DPI). Aranday-Cortes et al. (80) also noted upregulation of *IL-17A* mRNA in lymph node lesions 13 WPI, in comparison to control lymph node tissue. This upregulation occurred at each granuloma stage investigated. Notably, as granulomas developed, expression decreased until there was significantly less *IL-17A* in more advanced compared to early-stage lesions.

In addition to increased *IL-17A* gene expression, increased protein production has also been observed in *M. bovis* infection. McGill et al. (87) observed significantly higher levels of PPD_b_/EC-stimulated IL-17A protein secreted by PBMCs from experimentally *M. bovis-*infected cattle between 3 and 6 WPI. Increased numbers of antigen-specific IL-17A-secreting cells have also been found in blood from infected animals, with CD4^+^ T cells discovered to be the prominent source of IL-17A following stimulation. Similar findings in cattle were also reported by Steinbach et al. (88). Waters et al. (81) investigated IL-17A protein in whole blood and mRNA from PBMCs of experimentally infected cattle and observed a >9-fold upregulation post-infection, with correlations between gene expression and protein release, and between IL-17A and IFN-γ production. Moreover, higher IL-17A concentrations at 2.5 WPI correlated with increased lesion severity and mycobacterial burdens in cattle. Using stimulated PBMCs, Xin et al. (89) also observed significantly higher PPB_b_-stimulated IL-17A mRNA and protein in naturally and experimentally infected cattle compared to uninfected controls. The marked IL-17A responses elicited by *M. bovis* in cattle, combined with correlations to bTB pathology, point to the utility of IL-17A as a promising indicator of bTB disease progression.

### CXCL9

The chemokine CXCL9, also known as monokine induced by IFN-γ (MIG), has been reported as a mediator of the bovine anti-mycobacterial response during bTB, with a proposed role in attracting activated T cells, and granuloma development or maintenance (76, 77). Aranday-Cortes et al. (92) investigated *CXCL9* expression in lymph node granulomas of experimentally *M. bovis-*infected cattle. The early-stage granulomas showed significantly upregulated expression compared to the control tissue, followed by a significant decrease in *CXCL9* in early to advanced granulomas as lesions progressed. In contrast, Palmer et al. (77) studied pulmonary granulomas at 150 DPI and observed overall high and significantly elevated *CXCL9* expression compared to non-lesion lung tissue; however, the expression did not differ significantly between different granuloma stages. The differences in these two studies were attributed to tissue type, among other factors (77, 134). Klepp et al. (25) investigated *CXCL9* expression in PBMCs from naturally *M. bovis*-infected cattle and significant differences were observed between infected and healthy animals. Similarly, Palmer et al. (5) also observed significantly elevated *CXCL9* gene expression and protein levels in *M. bovis-*challenged cattle in response to EC/Rv3615c or PPD_b_ antigens, with detectable *CXCL9* responses by 2 WPI, which remained consistently and significantly higher than that of the control group.

In lions, EC-stimulated blood was used to assess *CXCL9* expression, and significantly increased levels of *CXCL9* enabled discrimination between *M. bovis-*infected, -exposed, and uninfected lion cohorts (83, 101). Also using EC (QFT) stimulated blood, Higgitt et al. (102) and Kerr et al. (103) were able to detect *M. bovis* immune sensitization by upregulation of *CXCL9* in spotted hyenas (*Crocuta crocuta*) and cheetahs (*Acinonyx jubatus*), respectively. In addition, Roos et al. (100) was able to show that upregulation of *CXCL9* could distinguish between *M. bovis-*infected and uninfected warthogs.

Overall, studies on *CXCL9* have demonstrated high levels of expression in tuberculous lung and thoracic lymph nodes, in addition to stimulated whole blood, in cattle and other species infected with *M. bovis*. The CXCL9 responses display a robustness akin to that of the IP-10 biomarker, although without the confounding effect of spontaneous production such as that of IP-10 in unstimulated samples (5).

### IL-10

The Th2-associated cytokine IL-10 is a critical anti-inflammatory mediator of innate and adaptive responses to pathogenic mycobacteria (35). The function of IL-10 is to deactivate macrophages and decrease production of reactive nitrogen and oxygen species; hence, in its absence, a stronger Th1 immune response is incited, while high levels of IL-10 are associated with increased susceptibility to mycobacterial infection (77, 135).

There appears to be an inverse relationship between IL-10 and IFN-γ. Welsh et al. (35) analyzed PBMC cytokine mRNA of experimentally infected cattle, and reported high IL-10 levels prior to infection, which gradually declined following infection as higher *IFNG* expression was detected. However, there was a sharp increase in IL-10 at 26 WPI, with levels higher than those pre-infection, in cattle that showed the greatest severity of disease. In addition, this was correlated with decreasing CMI and increasing humoral responses (35).

Similar patterns were seen in IL-10 expression in tissues. Widdison et al. (37) studied IL-10 expression in lymph node tissue, noting a significant decrease in IL-10 and an increase in the *IFN-*γ/IL-10 ratio in infected compared to uninfected cattle. There was also a significant negative correlation between lesion scores and mycobacterial load in lymph nodes and IL-10 expression. These results are similar to those from Thacker et al. (76) who also compared IL-10 between a high and low pathology groups of *M. bovis*-infected cattle in which they discovered two-fold lower IL-10 expression in the high pathology cohort. However, these two studies were conducted at 16 and 18 WPI. Considering the role of IL-10 in limiting tissue destruction, hence smaller and less necrotic lesions displaying the highest IL-10 levels, these results were unsurprising and could explain the later peak (26 WPI) observed by Welsh et al. (35). In the Widdison et al. study, the combined observation of suppressed IL-4, IL-10, IL-6, and TNF in the infected group, together with maintenance of IL-12 and IFN-γ levels, suggested that suppression was specific and not just a general consequence of infection and necrosis from a developing chronic response.

Interestingly, Blanco et al. (96) did not observe any downregulation of IL-10 in PBMCs from infected cattle with lesions, and the expression level was similar to that of infected animals without lesions. Similarly, Palmer et al. (77) observed no difference in IL-10 expression between granulomas and non-lesioned lung tissue, noting that the low levels observed were expected of active granulomas. However, when combined with IL-2 and IL-17 in a predictive biomarker combination, IL-10 enhanced the classification of infected/lesion-negative animals and was hence acknowledged as a potential identifier of disease progression in herds with no clinical signs of bTB (96). The ratio IFN-γ/IL-10 has also been acknowledged as a potential indicator of *M. bovis* disease severity in red deer (95). However, most studies used a single time point to assess IL-10 expression, which may not reflect the dynamic levels of cytokines during granuloma formation. In support of this, Canal et al. (94) observed significantly higher IL-10 expression in more advanced (stage III and IV) granulomas of lymph nodes and lung, compared to stages I and II, in naturally *M. bovis* infected cattle. Finally, lung lesions and respiratory lymph nodes from goats, experimentally infected with *M. bovis*, revealed high levels of IL-10 and highlighted the important role of this cytokine in granuloma formation (97).

### IL-22

The cytokine IL-22 is part of the IL-10 family and is produced by natural killer, mast and T cells, predominantly cell types Th17 and Th22 (80). Together with IL-17A, IL-22 has emerged as a critical effector cytokine required for the detection and clearance of bacilli in TB studies, however, its role in bTB is less studied. It has been shown to induce protection and may inhibit mycobacterial growth inside macrophages (136).

Aranday-Cortes et al. (80) investigated IL-22 mRNA in a murine bTB model, followed by a study in PBMCs from infected cattle, and observed a 74-fold upregulation of IL-22 in the lungs of infected compared to naïve mice and highly significant upregulation in the PPD_b_-stimulated PBMCs of the infected cattle. The predominant source of IL-22 was CD4+ T cells, similar to IFN-γ. Ray Waters et al. (90) and Steinbach et al. (88) confirmed these observations both at the gene and protein level, respectively, with the latter using naturally *M. bovis-*infected cattle. Palmer et al. (5) also observed upregulated IL-22 expression in infected cattle, at 5, 8, 12, and 16 WPI, compared to uninfected controls.

Another study by Aranday-Cortes et al. (92) examined tuberculous granulomas from infected cattle. The study reported upregulation of IL-22 in bTB lymph node lesions with a clear trend of decreasing mRNA expression from granulomas in early to advanced stages, indicating the potential of IL-22 as a biomarker for bTB pathology. Rusk et al. (91) also noted a lack of IL-22 expression by T cells within late-stage granulomas from lung and mediastinal lymph nodes, confirmed by Palmer et al. (93) who observed very low expression levels of IL-22 in lymph nodes with advanced granulomatous lesions, i.e., samples collected at ±21 WPI, with no differences in expression between granulomatous and uninfected lymph nodes. Klepp et al. (25) studied IL-22 expression in PBMCs from naturally infected cattle and in addition to observing upregulation of IL-22 in the infected group, also found that IL-22 could significantly differentiate *M. bovis* infected cattle with either negative TST or IGRA results from uninfected animals. Hence, IL-22 may be useful as an ancillary biomarker for bTB detection where the results from the TST and IGRA fail to detect infected animals.

## Discussion

This review describes host CMI biomarkers with diagnostic potential for the detection of *M. bovis* infection and bTB, with a focus on more recent research and knowledge gaps, especially as these pertain to wildlife species. The CMI response is a vital component of host adaptive immunity to *M. bovis* (34, 35). Cattle and badger studies have demonstrated the involvement of the major T lymphocyte types and production of CMI-based cytokines and chemokines during *M. bovis* immune response development, accompanied by a shift from the predominant pro-inflammatory, Th1-biased response to more anti-inflammatory, Th2 functions as infection develops (36, 37, 51, 94). With a focus on the observation and measurement of CMI responses, detected through changes in gene expression, protein release or both, promising diagnostic biomarker targets and their associated limitations for multi-species application have emerged.

The choice of CMI-based methods for diagnostic purposes is in part motivated by the early and specific response that is elicited in most *M. bovis* host species studied to date (51). The delayed type hypersensitivity reaction measured *in vivo* by the TST is mimicked in stimulated blood cultures and can therefore result in a higher Se and Sp, due to the controlled *in vitro* conditions and parameters, for *M. bovis* detection (43, 58). Additionally, the specific cytokines and chemokines, produced during the adaptive immune response, that are detected by these assays is a feature applicable to most (if not all) mammalian species, thus allowing for translational use across species. Further advantages of *in vitro* CMI assays include even earlier detection times of an immune response than the TST (as early as 1-week post infection), only a single immobilization or handling event to collect blood samples, and more potential for the standardization of tests and reagents without in-field variation and operator bias (69). Moreover, the cost differences between *in vivo* (TST) and *in vitro* CMI assays may be over-estimated and CMI tools could prove more cost-efficient than assumed, with further cost reductions anticipated by increased use allowing the scale-up of production, and the automation of assays (such as ELISA kits) that lower laboratory costs (13).

The most used cytokine biomarker for TB diagnostic assays is IFN-γ, a critical Th1 cytokine produced upon lymphocyte activation in defense against *M. bovis* (43). This cytokine is also vital for the formation and function of granulomas, a hallmark of the response to *M. bovis* in host species (94, 120). Compared with other cytokine assays to date, IGRAs are robust and comparatively easy to standardize, and the development of new IGRAs for multiple non-bovine species has been suggested for bTB screening and control (13, 120). The increasingly widespread use of IGRAs (such as Bovigam®) in cattle, due to US- and EU-approval as a TST adjunct for parallel testing in bTB eradication programs to increase Se, may also explain interest in its use for wildlife species. Although IGRAs have been successfully applied to several domestic and wildlife species, including but not limited to cattle, goats, cervids, buffalo, white rhinoceros, and wild dogs, there have been limitations encountered when using them in other *M. bovis* host species (43, 111, 113, 114, 137). Aside from technical challenges related to the lack of available diagnostic tests and reagents for most wildlife, this may also be due to species heterogeneity in predominant immune response pathways to *M. bovis* (45, 51). Although *M. bovis* was first strongly associated with cattle, this pathogen has adapted and evolved to infect a broad range of animal host species, which may present with shared or unique characteristics in their immune responses (4, 9, 120). Although less studied, there may be differences in the IFN protein structure or amount produced between species, resulting in decreased expression and thus detection of this cytokine as observed in lions and warthogs (83, 98). Another difference between species is disease susceptibility together with infection pressure in the specific environment, hence, some animals could be exposed to high pathogen doses leading to rapid disease progression, or lower levels resulting in sub-clinical infection (137). The disease state and dose-dependent immune response may also influence the patterns of cytokines detected, as observed in experimentally infected badgers that showed consistent CMI responses with a high dose of *M. bovis* yet those with subclinical presentation had weak CMI responses, although with no effects on the humoral response (51). This could explain the more efficient use of alternative biomarkers or methods observed in species such as lions and warthogs, in which IFN-γ detection appeared less optimal (45, 47, 83, 98). However, biomarker signatures associated with a subclinical bTB state in animals have not yet been investigated, in contrast to human studies (138). A final factor to consider is the influence of *M. bovis* strains on host immune responses. Although it is acknowledged that the virulence of pathogenic mycobacteria is linked to genetic and phenotypic characteristics, this has only been confirmed for *M. tuberculosis*, aided by in-depth research using animal models (120, 139). Hosts of *M. bovis* are typically outbred species, thus, discerning the influence of *M. bovis* lineage from host heterogeneity is challenging. However, virulence variations between strains have been reported that suggest potential correlations between strain genetic differences and bTB development (139, 140).

In addition to a dominant early response, changes in IFN-γ responses have been correlated to pathological changes, although an IGRA to distinguish between *M. bovis* infection and bTB disease has not yet been developed (84, 89). Considering the correlations observed between *IFNG* expression and granulomatous lesion development, the quantitative measurement of IFN-γ in different cohorts of defined bTB states from early infection to active disease could enable the development of an IFN-γ cytokine assay to differentiate *M. bovis-*infected from diseased animals. However, species specific validation would be critical due to observed differences in *IFNG* expression profiles during bTB progression, as observed between fallow deer and cattle (93, 94, 123). In light of the potential drawbacks to IFN-γ detection, including sample handling that necessitates a short period between blood collection and processing and variable test performance parameters depending on species and disease stage, alternative cytokines, and chemokines to IFN-γ have emerged as additional tools for *M. bovis* infection and bTB diagnosis.

Pro-inflammatory cytokines are released early after *M. bovis* infection and are thus expected to be promising candidates for biomarkers of infection due to the predominant Th1 response observed in several host species. In addition to IFN-γ, two major pro-inflammatory cytokines are IL-1β and IL-6, although not much more than a role in general mycobacterial infection is known for the latter. The former, IL-1β, has demonstrated high Se for *M. bovis* detection in cattle, particularly when used in parallel with an IGRA (5, 31). Although not explicitly pro-inflammatory, two biomarkers (IP-10 and IL-8) have been detected at higher concentrations than IFN-γ and have shown possible correlation with bTB progression in cattle and African buffaloes (5, 72, 84). Considering their robust response early after *M. bovis* infection, these biomarkers could be well-suited for detecting *M. bovis* infection in less studied species. Another cytokine that demonstrates robust levels from an early infection stage is CXCL9, responsible for CD4^+^ lymphocyte recruitment (5, 100, 101).

There are limitations associated with CMI-based diagnostics that include reduced Se due to anergy (reduced detectable Th1-biased CMI responses as bTB progresses), and interference from co-infection and vaccination (120). However, the application of a parallel testing scheme, whereby at least two different tests able to detect slightly different sub-populations of infected animals (i.e., animals at different stages of infection) are used together, has shown promise in combatting these drawbacks, particularly in *M. bovis-*endemic settings (69, 111). The demonstration of two dominant pro-inflammatory cytokines, IFN-γ and IL-1β, that increased Se without compromising Sp when diagnosing *M. bovis* infection in cattle, indicates that even slight differences in cytokine pathways and functions can provide a testing scheme to improve detection of the maximum number of infected animals. Moreover, for species in which IFN-γ detection is problematic, the detection of additional or alternative pro-inflammatory cytokines could aid the early detection of *M. bovis*. Therefore, considering the robust levels of IL-8 and its good agreement with both the TST and IGRAs, this marker could also prove a promising option for enhancing *M. bovis* detection. Another example of parallel application is the measurement of IP-10 with IFN-γ that has enhanced Se in buffaloes and cattle, with high IP-10 production having a particular advantage in very early stages of infection (29, 30). Moreover, Klepp et al. (25) demonstrated the utility of IL-22 for the diagnosis of *M. bovis* infections that both the TST and IGRA failed to detect, not unexpected from a cytokine marker that stems from a separate and recently described T cell lineage, Th17.

Infection with *M. bovis* typically results in an early Th1 bias that tends to decline as the Th2 response increases, with shifts from Th1 to Th2 responses showing correlations to increased pathology (94). Hence, whether the objective is optimum detection of all infected animals, the detection of highly infectious individuals (i.e., animals that are shedding *M. bovis* bacilli) or the distinction between infection and active disease, it is expected that a diagnostic algorithm would benefit from using a combination of pro- and anti-inflammatory biomarkers. Moreover, the immunological response to *M. bovis*, in terms of bTB disease progression, is a dynamic process and non-linear (77). Thus, different biomarker signatures could be used to identify bTB progression, as suggested by Blanco et al. (96), Kelley et al. (56), and Palmer et al. (5). One example, shown by immunohistochemical analysis of granulomatous lesions of lymph nodes and lung in cattle, is the combination of pro-inflammatory IFN-γ and IL-1β with IL-10 (94). The two pro-inflammatory cytokines demonstrated contrasting yet equally significant associations with granuloma development and lesioned vs. non-lesioned tissue, highlighting the diversity of individual cytokine functions. The study also observed a lack of IL-10 expression in advanced granulomas that correlates with previous findings of progressively decreasing IL-10 in cattle with severe pathology (76, 94). The anti-inflammatory cytokines IL-4 and IL-10, which typically present with inverse correlations to IFN-γ over the course of infection, have shown good potential in distinguishing disease states; thus, their inclusion is suggested for biomarker signatures that cover both early and late stages of infection and bTB, with the potential to enhance diagnostic Se or Sp particularly for enhanced detection of infected animals (37, 75, 78, 94, 95). A panel of biomarkers could also provide a promising method for wildlife species in particular, due to the measurement of varied immune responses from a single sample that would provide greater confidence in the animals' disease state without the need for repeat testing, a significant challenge for most wildlife testing. Alternative potential biomarkers of bTB disease progression include IL-17A, also of the Th17 subset, and CXCL9 due to the observed increase as infection progresses with additional correlations to lesion severity (25, 81). This highlights the utility of adding biomarkers from different T cell subsets in a bTB testing scheme and warrants their investigation in wild animal species, especially considering the infeasibility and lack of standardization of the TST outside of common domestic species.

Due to the recognized role of wildlife in the maintenance of *M. bovis*, research on the development of assays for wild species (partially or fully validated) is increasing. A significant challenge in the development of CMI biomarkers for *M. bovis* in wildlife is the difficulty in obtaining sufficient reference samples to investigate and validate diagnostic assays. The lack of validated *ante mortem* tests makes diagnostic tests even more challenging to perform under field conditions, particularly in endemic settings (19, 56). Furthermore, the procurement of gold standard reference cohorts remains a trade-off between obtaining reliable samples and the loss of valuable animals, particularly for negative cohorts, that are required to obtain data from *post mortem* examination and mycobacterial culture (43). There are statistical tools that provide alternatives to the use of reference standards, such as latent-class or Bayesian approaches; however, statistical methods require large sample sizes, among other limitations (19, 21, 71). In acknowledgment of these challenges, recent policy (in the form of additional chapters for the OIE Terrestrial Manual) was adopted between 2014 and 2016, including statistical approaches to validation, that extends to the validation standards for diagnostic tests to wildlife (Chapter 2.2.7., OIE Terrestrial Manual 2018). Two validation pathways are provided that allow provisional recognition of a test if the complete validation process is hindered. This agrees with the chapter's emphasis on fit-for-purpose assays, particularly as diagnostic testing objectives may differ between domestic animal and wildlife species. The chapter also recognizes the difficulties in obtaining wildlife reference standards (particularly negative cohorts), suggesting latent-class models for performance estimates in lieu of a perfect (gold standard) reference (141). On the other hand, the imperfections of the gold standard requirement for mycobacterial culture have been acknowledged more recently; hence, alternative definitions for reference cohorts that improve test performance parameters may also be applicable (13, 51, 69).

The successful use of IGRAs and other cytokine assays in wildlife depends on the availability (and costs) of suitable tests or reagents. However, commercial CMI biomarker detection platforms are being developed and optimized using common domestic species with unknown cross reactivity to wildlife species. Therefore, researchers may need to develop novel species-specific reagents and assays, although these would have a very limited market. However, if cross reactivity can be determined using commercial reagents, this has the advantage of ready access by other researchers without having to exchange reagents. Indirect ELISAs with cross-reactive reagents have facilitated their use in related species, such as the application of bovine ELISAs to African buffaloes (32, 111) or equine ELISAs in white rhinoceros (113). However, another viable alternative is cytokine gene expression assays that can be adapted or developed for use in closely related species and even extended to non-related species if the sequences are conserved, which has been demonstrated for cytokine sequences in wildlife species (26, 142). Gene expression assays have been used in less common species such as warthogs, hyenas, and lions (83, 98, 102). The development of cytokine and chemokine multiplex assays that detect several targets simultaneously in a single sample (of small volume), with options for customization, has also aided the development of biomarker signatures (13, 143). Recently, the MILLIPLEX® bovine cytokine/chemokine platform demonstrated detection of several novel protein markers in buffaloes, with the aim to enhance *M. bovis* detection and potentially differentiate bTB disease states with the use of multiple diagnostic markers in this species (112). Transcriptome analysis has also been conducted using *in vitro* murine bTB models and PBMCs from cattle to identify more comprehensive gene panels involved in responses to *M. bovis*, although correlations between signatures and a potential incipient vs. advanced bTB state remain an avenue for future research (80, 144). Considering the multiple challenges faced when validating a single new CMI-based diagnostic assay, including the limited funding and research on candidate biomarkers in wildlife, the lack of progress regarding multiple marker signatures is not surprising. Moreover, the costs and feasibility of performing these assays for a range of species should be considered when prioritizing the development of biosignatures for wildlife bTB diagnosis.

The success of management and control strategies for bTB is only as effective as the diagnostic assays it relies upon. The use of CMI cytokine and chemokine biomarkers has already improved insight on the comparative immunology of *M. bovis-*infected hosts, with experimental and natural infection studies conducted in cattle, badgers and cervids, among other species. Moreover, understanding the contribution and role of dominant profiles, such as the Th1/Th2 responses, to the development of pathology aids the identification and development of diagnostic biomarkers and biomarker panels. However, despite progress in understanding *M. bovis*-induced immune responses, the research and diagnostic biomarkers described here are still primarily restricted to cattle. In comparison to domestic species, limited resources are allocated to studies of bTB in wildlife, resulting in a paucity of information on *M. bovis* infection and disease development in other naturally infected hosts (43, 51). Hence, more species-specific research is required, together with the development of standardized, multi-species tests and reagents, and investigation of candidate biomarkers in alternative samples types, i.e., serum, due to the practical, in-field and diagnostic potential of circulating responses. CMI-based assays may also be further improved with the addition of enhanced, immunodominant antigens for stimulation (in addition to ESAT-6 and CFP-10) to increase test Se and Sp. Additionally, advances in techniques from multiplex biomarker detection platforms to powerful statistical approaches that estimate population characteristics when true disease status is unknown, or when logistical challenges prevent acquisition of gold standard reference cohorts, will further enable the validation of enhanced immunological tools in both domestic animals and wildlife.

## Author Contributions

KS, MM, and WG conceived the idea for the review. MM and RW provided funding. KS, MM, LK, WG, and RW contributed to the drafting and editing of the manuscript. All authors reviewed the manuscript.

## Conflict of Interest

The authors declare that the research was conducted in the absence of any commercial or financial relationships that could be construed as a potential conflict of interest.
